# Perceived value of video games, but not hours played, predicts mental well-being in casual adult Nintendo players

**DOI:** 10.1098/rsos.241174

**Published:** 2025-03-12

**Authors:** Nick Ballou, Matti Vuorre, Thomas Hakman, Kristoffer Magnusson, Andrew K. Przybylski

**Affiliations:** ^1^ Oxford Internet Institute, University of Oxford, Oxford, UK; ^2^ Tilburg University, Tilburg, The Netherlands; ^3^ Karolinska Institute, Stockholm, Sweden

**Keywords:** video games, digital trace data, well-being

## Abstract

Studies on video games and well-being often rely on self-report measures or data from a single game. Here, we study how 703 casually engaged US adults’ time spent playing for over 140 000 h across 150 Nintendo Switch games relates to their life satisfaction, affect, depressive symptoms and general mental well-being. We replicate previous findings that playtime over the past two weeks does not predict well-being, and extend these findings to a wider range of timescales (1 h to 1 year). Equivalence tests were inconclusive, and thus we do not find evidence of absence, but results suggest that practically meaningful effects lasting more than 2 h after gameplay are unlikely. Our non-causal findings suggest substantial confounding would be needed to shift a meaningful true effect to the observed null. Although playtime was not related to well-being, players’ assessments of the value of game time—so-called gaming life fit—were. Results emphasize the importance of defining the gaming population of interest, collecting data from more than one game, and focusing on how players integrate gaming into their lives rather than the amount of time spent.

## 1. Introduction

Video games played on smartphones, computers, or home consoles are now a widely pursued form of leisure that involves social interaction, creativity, problem-solving and growth [1]. Major firms like Nintendo have sold hundreds of millions of game consoles in recent years [2] and online platforms such as Steam regularly attract upwards of 30 million players online at any given time [3]. This staggering investment of human attention and behaviour has inspired both national [4] and international [5] health bodies to focus on play as a potential contributor to psychopathology.

The extent to which games might be understood as behaviorally addictive remains hotly debated [6–8] and the broader scientific conversation has increasingly focused on how not just quantity, but also quality of play relates to player health. Although there is increasing recognition that not all screen time—or in the case of games, playtime—is created equal [9], it remains a major research focus. Research has identified a range of factors that co-determine how time spent with games relates to health: for example, aspects of a game’s design such as its social affordances [10], the context of when and where one plays [11] and players’ motivation [12].

In matters of health policy, overall time spent with games—regardless of what or why one plays—remains central to how games are thought to influence player outcomes. Parental control tools on platforms like Xbox and PlayStation foreground time limits as a primary means of enforcing healthy gaming behaviour [13]; for adults, a growing array of self-control tools, apps and dashboards offer the ability to ‘limit or cut yourself off from apps and games’ (https://focusme.com), saving users ‘1.23 h’ (https://www.opal.so) or ‘up to 2.5 h a day’ (https://freedom.to/). News media suggest time-based limits (e.g. [14]), often referencing the since-abandoned 2 × 2 rule from the American Academy of Pediatrics: no screen time for children under two, and no more than 2 h per day for children older than two [15]. Likewise, the American Psychiatric Association’s description of Internet gaming disorder characterizes pathological engagement with games, in part, as involving ‘8−10 hours or more per day [and or] least 30 hours per week’ [4, p. 796]. On a larger scale, time-based limits such as Korea’s 10 year Youth Protection Revision Act prohibited young people from playing games between 00.00 and 06.00 [16]. More recently, China put in place a weekly 3-h limit for those under the age of 18 [17]. The effectiveness of such regulatory steps has been challenged [18,19].

A better understanding of how time spent with games relates to players’ health, for good or ill, is needed. Given that play takes many forms and happens across many different games, researchers greatly benefit from access to digital trace data—histories of user actions generated when interacting with technologies such as a game or online platform. Digital trace data can provide much greater detail about what, when and how much people play than is possible in self-report data, which consistently show substantial discrepancies compared with digital trace data collected by online platforms [20] and independent researchers [21] alike. Previous studies on games found that an additional hour of *Animal Crossing: New Horizons* trace data predicted just a 30 min increase in self-reported play—a nearly 50% discrepancy [22]—and that *Everquest 2* players’ estimates correlated only *r* = 0.37 with logged estimates, with underestimates slightly more common than overestimates [23].

Only a handful of studies have applied digital trace data to the study of games and well-being [12,22,24] (see [25]), in part because these data can be very difficult to acquire: researchers must build or rely on unstable technical systems to log data themselves or negotiate individual agreements with games companies who have historically been reluctant to share data [26,27]. Where digital trace data have been collected, however, results have been informative. Brühlmann *et al*. [12] used playtime and in-game behaviour to identify subgroups of *League of Legends* players who had more negative in-game experiences. Johannes *et al*. [22] looked at playtime in *Animal Crossing: New Horizons* and *PvZ: Battle for Neighborville* and found a positive but likely negligible correlation. A follow-up study expanded this to seven games, finding that changes in playtime over the course of six weeks were unlikely to affect subsequent well-being [25]. Larrieu *et al*. [24] follow high-intensity *Rainbow 6: Siege* players and find no link between playtime and quality of life across any identified player types.

Though informative, this early work has a vital scope limitation: digital trace data were only available for a single game for each player–participant. It was not possible to know what other games a participant was, or was not, electing to play. Players regularly switch between games over the course of a day or week based on mood, available time and social context [28,29]; data collected for one particular game may thus tell us little about *overall* playtime or its relation to well-being.

An important frontier for the field, therefore, is to collect holistic digital trace data that reflect behaviour in not just one game, but all games played (which may include various games on one platform, such as Nintendo Switch or Steam, or all games played across several platforms a player uses). At present, our understanding of even basic phenomena such as the true volume of play in different demographics relies on the same inaccurate self-report data, itself often provided by market research firms using opaque methodologies. Capturing play at the platform level represents one step towards this goal of studying a player’s complete gaming diet. To our knowledge, the only study to investigate player health with platform-level digital trace data is [28], which found no meaningful relationships between Xbox playtime and well-being over three months. The current study complements this earlier work by investigating how well-being relates to play on an alternative platform, Nintendo Switch, which has a more casual audience and a distinct library of games, and by analysing a longer window of play behaviour spanning up to 1 year.

### 1.1. Present study

In the present research, we report on a study in which we independently recruited a large sample of adult video game players, surveyed them about their motivation and well-being, and joined these responses to digital trace data on Nintendo Switch video game play, which was provided via a data-sharing agreement with Nintendo of America. Our central aim was to test the extent to which the amount of time participants spent playing related to their psychological well-being. Our analysis plan was pre-registered at https://osf.io/sjqyt.

More specifically, our first hypothesis was to test whether the null relations reported in earlier work [22,25,28] would replicate in an independent sample of Nintendo Switch play. In our first hypothesis, we predicted that there would be no practically significant association between video game playtime in the previous two weeks and life satisfaction (H1a), affect (H1b), depressive symptoms (H1c) or general mental well-being (H1d).

We were also interested in understanding how relationships between play and well-being might depend on the choice of what timescale of play researchers consider relevant to investigate. To this end, we examined relationships between well-being and a wide range of time windows of play preceding the well-being question. While pre-registered, the large number of models implicated in our analysis plan is prohibitively large enough to be aptly framed as a narrow hypothesis test. We, therefore, operationalize this as an exploratory research question: how does the relationship between playtime and well-being differ across different gaming observation windows ranging from 1 h to 1 year?

Lastly, we investigated what factors might moderate the relationship between playtime and well-being. To test this exploratory question, we assessed potential moderation by participant gender and age, as well as a subjective sense of how players thought gaming had positive or negative relationships to various life domains such as work, relationships, school performance, and social health.

## 2. Method

### 2.1. Design and recruitment

**Figure 1.**
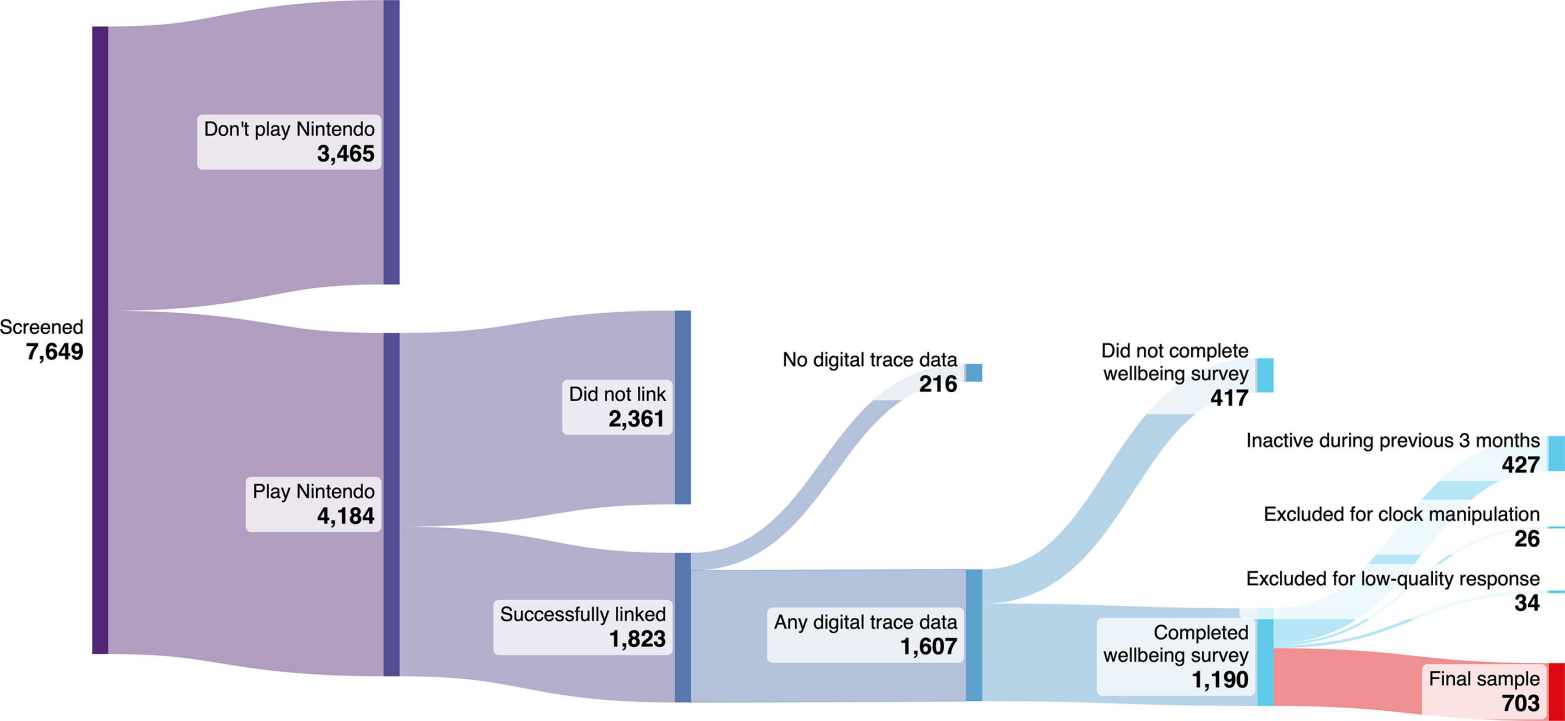
Participant flow, from recruitment to final sample.

Our participant flow is shown in figure 1. We recruited participants from Prolific, a participant recruitment platform, who were: (1) at least 18 years old, (2) proficient in English, (3) residents of the USA and (4) active video game players (self-defined, based on *a* > 0 response to Prolific’s built-in screening item ‘How many hours per week do you play video games on average?’). We first distributed a screening questionnaire to 7649 participants asking which video game platforms they were active on; of these, 4184 indicated that they played games on Nintendo Switch. Participants who played on Nintendo Switch were active on 1.8 additional platforms on average (e.g. also using Playstation and iOS); Nintendo playtime therefore captures only an unknown portion of overall gaming activity. We return to this limitation in §4.

We invited these participants to a second survey wherein they retrieved an account identifier from the Nintendo Web interface using the events QR code, available at https://accounts.nintendo.com/qrcode, and they shared these unique identifiers with us. This identifier is separate from their username, and cannot be used by anyone besides Nintendo—including our research team—to link the player to personally identifiable information. A total of 1823 participants completed the linking process. We sent the account identifiers to Nintendo of America, who in turn sent us each player’s pseudonymized play history from 1 May 2022 to the present. Data collection began with a pilot study of 100 participants on 15 November 2023, which was combined with primary data collection from 12 February 2024 to 6 May 2024.

Of the participants who completed the linking process, 1607 had eligible Nintendo data (i.e. any play sessions of a first-party game—a game published by Nintendo or one of its subsidiaries, as opposed to a third-party publisher—from 1 May 2022 to present). These 1607 were subsequently sent a well-being survey in Qualtrics detailed below. The well-being survey was completed by 1191 participants; our pre-registered stopping rule went into effect when five or fewer participants per day completed the survey for three consecutive days.

As pre-registered, we excluded 427 individuals who had no playtime during the previous 3 months, indicating that they are not active Nintendo players, and 26 people who logged more than 24 h of playtime on any single day or who had a session that took place in the future, indicating a technical problem or manipulation of the system clock for in-game benefits. We further excluded 34 participants who displayed signs of careless responding (e.g. so-called straight lining or seemingly random responses). In total, we excluded 487 participants (some participants were excluded on multiple grounds), leading to a final sample of 703.

Participants were paid £0.15 for the 1 min screening questionnaire, £3 for linking their data plus a £2 bonus payment if data were successfully retrieved and £4 for a 22 min well-being survey. This study received approval from the University of Oxford Social Sciences and Humanities Interdivisional Research Ethics Committee (OII_C1A_23_107).

### 2.2. Participants and exclusions

Participant demographics are shown in table 1.

**Table 1.** Participant demographics.

descriptor	variable	value
age	mean (s.d.)	31.5 (7.7)
	median (IQR)	31.0 [26.0;36.0]
	min./max.	18.0/68.0
gender	man	376 (53.5%)
	woman	267 (38.0%)
	non-binary or other gender identity	60 (8.5%)
employment status	working full-time	338 (48.1%)
	working part-time	120 (17.1%)
	other employment status	97 (13.8%)
	not currently employed	91 (12.9%)
	student	57 (8.1%)
ethnicity	White	456 (64.9%)
	Asian	82 (11.7%)
	Mixed	75 (10.7%)
	Black	54 (7.7%)
	other ethnicity	36 (5.1%)

### 2.3. Measures

Participants completed a self-report survey that took on average 22 min to complete. The survey included background factors such as demographics and life circumstances, a series of cognitive tasks, as well as self-report measures of time use and motivations for video game play. In this paper, we focus on the playtime data and the self-reported well-being measures and detail only those measures below. Other measures were intended for analyses beyond the scope of the current paper. All study data and materials are available on the OSF (see electronic supplementary materials).

*Video game playtime* was measured by collecting a record of each player’s individual game sessions for all first-party video games played on a Nintendo Switch. These data were provided by Nintendo of America. Playtime was calculated by summing the duration of all (partial) sessions that occur during a given time period relative to the participant’s survey response, based on the logged session start and end times. For ease of interpretation, playtime in all observation periods longer than 24 h is reported as mean minutes of play per day. It is important to note that these data included only digital trace data for games published by Nintendo and its close business partners (e.g. The Pokémon Company), but not games made by third-party publishers (e.g. Electronic Arts). Nintendo-published games accounted for 63% of all playtime across our sample; the 37% of play data from third-party games are therefore treated as missing. We return to this limitation in §4.

*General mental well-being* was measured with the WEMWBS [30]. Players rated 14 statements about how they felt during the past 2 weeks such as ‘I’ve been dealing with problems well’ and ‘I’ve been feeling good about myself’ on a five-point scale from 1 (‘none of the time’) to 5 (‘all of the time’). Scores were calculated by taking the mean of all items.

*Depressive symptoms* were measured with the PROMIS Short Form 8a [31]. Players rated eight statements about how they felt in the past seven days such as ‘I felt hopeless’ and ‘I felt I had nothing to look forward to’ on a five-point scale from 1 (‘never’) to 5 (‘always’). Scores were calculated by taking the mean of all items.

*Life satisfaction* was measured with the one-item Cantril self-anchoring scale [32]. Participants were prompted with ‘Please imagine a ladder with steps numbered from 0 at the bottom to 10 at the top. The top of the ladder represents the best possible life for you, and the bottom of the ladder represents the worst possible life for you. On which step of the ladder would you say you personally feel you stood over the past two weeks?’ Participants responded on a scale from 0 to 10, which was rescaled to continuous values between 1 and 5 to match the range of WEMWBS and PROMIS measures.

*Affect* was measured with a single item: ‘How are you feeling right now?’ [33]. Participants responded using a 100-point visual analogue scale (VAS) with endpoints ‘very bad’ and ‘very good’, which we rescaled to continuous values between 1 and 5 to match the WEMWBS and PROMIS measures.

*Gaming life fit* was measured with a draft measure asking players to rate the contribution of gaming to five life domains (work/school, social participation, cognitive health, emotion regulation and daily routines) on a seven-point bipolar scale from ‘greatly interfered’ to ‘greatly supported’. We took the average of these to generate a formative indicator of the degree to which players perceive their gaming to be beneficial or harmful to other aspects of their lives. This measure has not been used or validated before, and we return to this in the discussion.

### 2.4. Analytic approach

To test H1, we fit multiple regression models with playtime over the previous two weeks as the primary predictor, all demographic variables as covariates (age, gender, highest level of education and employment status), and the corresponding mental health variable as the outcome. For example, for H1a (life satisfaction predicted by the previous two weeks of play), the model in R was


{{lm((lifesatisfaction)∼(playtimeintheprevioustwoweeks)+age+gender+education+employment)}}.

We applied a similar analysis approach to our exploration of H2 concerning other timescales; we primarily applied multiple regression with well-being predicted by playtime aggregated over various time periods and the same covariates but also explored potential nonlinear alternatives and moderation analyses (detailed below).

We interpreted the playtime coefficient estimates from these models in reference to pre-specified smallest effect sizes of interest (see below): if the 99% confidence interval is fully within the upper and lower equivalence bounds, this provides evidence to reject a practically meaningful association.

We conducted all statistical analyses with R version 4.3.2 [34]. We used an alpha of 0.01.

### 2.5. Smallest effect size of interest

We specify the smallest effect size of interest (SESOI) as a 1 h change in (daily) playtime associated with a 0.06-scale point change in mental health on a 1−5 scale, in line with Ballou *et al*. [28], who justified that value based on previous research on minimally important differences (approximately 0.3–0.4 scale points on a 1−5 scale for PROMIS and WEMWBS measures) and daily leisure time available to US adults (approx. 5 h; Sturm & Cohen [35]). Any association smaller than 0.06 indicates that the average person does not have enough time in the day to modulate their play to an extent that it would register a perceptible difference in their well-being.

Note this method of specifying an SESOI is predicated on a causal interpretation—it implicitly imagines a world where one can intervene on playtime (our predictor) and have an effect of a certain size on mental health (our outcomes). It is very unlikely that our cross-sectional analyses can provide unbiased causal estimates. Instead, our goal is to use associations to place boundaries on the size of a possible effect. In other words, if there is no meaningful correlation between playtime and mental health, there is even less likely to be a meaningful causal effect between playtime and mental health. We support this reasoning with simulations presented in the discussion.

## 3. Results

### 3.1. Descriptive results

**Figure 2.**
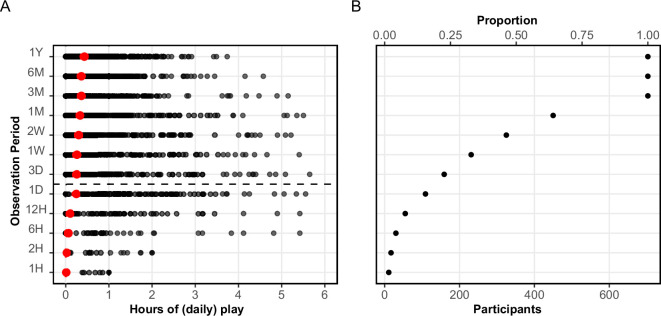
Description of playtime in the sample. Panel A shows the distribution of playtime across players for each observation period (time window leading up to the survey). Means are shown in red; hours of play for observation periods below the dashed line indicate total hours, whereas hours of play for periods above the dashed line reflect hours of play per day. Panel B shows the proportion of players who logged at least one play session in that period. Raw descriptive statistics are available in the electronic supplementary material (tables-figures/playtimeDescriptives.csv).

Given the lack of holistic or platform-level data available in the literature, our first goal was to simply describe the volume of play. This is visualized in figure 2, which shows that despite a total play volume of more than 140 000 h, our sample was largely minimally engaged with first-party Nintendo games. During the two weeks prior to survey completion, just over half of the sample had no sessions logged. The top 10% of players were moderately engaged, playing an average of 60 minutes per day. Sessions of a game lasted on average 41.9 min (10th percentile: 9.1; 90th percentile: 147.5).

The results of this study are therefore reflective of a largely casual population of players—at least with respect to Nintendo titles. We argue that this population is nonetheless an important one to study alongside the ongoing research focused on so-called hardcore players: if video games were to meaningfully affect well-being, we may expect a larger impact for people who rarely play but happen to play for 1 h, than for a highly engaged population of people who tend to play 3 h per day but happen to play 4 h. We return to this limitation on generalizability in §4.

### 3.2. H1: previous 2 weeks of playtime and mental health

We began by analysing H1, which concerned the relationship between mental health and the previous two weeks of playtime. This time period is common in the literature and served as a way to conceptually replicate a previous study focused on one game [22] using platform-level data.

**Figure 3.**
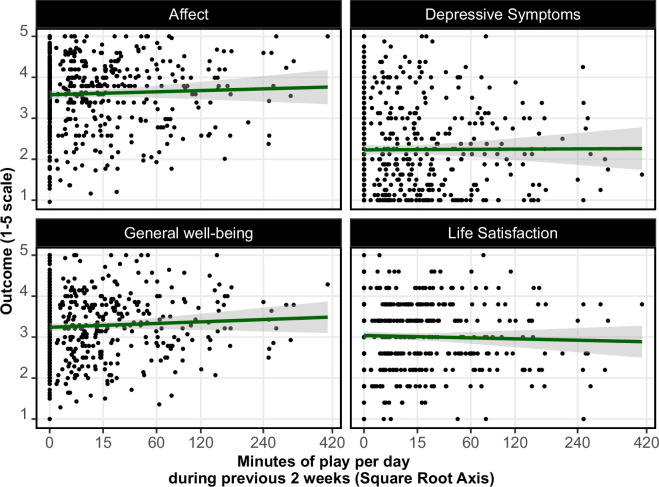
Scatterplots depicting the relationship between video game playtime during the previous two weeks (mean minutes of play per day) and four types of well-being.

Results are visualized in figure 3. Multiple regression models found no evidence that people who played 1 additional hour per day in the previous two weeks differed from their peers with regard to life satisfaction (*B* = −0.02; 99% CI [−0.12, 0.05]), affect (*B* = 0.08; 99% CI [−0.03, 0.19]), depressive symptoms (*B* = −0.06; 99% CI [−0.19, 0.07]) or general mental well-being (*B* = 0.08; 99% CI [−0.02, 0.18]).

However, due to lower-than-expected response rates and total volume of playtime, there is too much uncertainty around our estimates to confidently reject the presence of a meaningful relationship using our original SESOI of 0.06; following our inference criteria, the results of our original hypothesis tests are all inconclusive. We, therefore, interpret our results as indicating an *absence of evidence* for a relationship between playtime and well-being but do not conclude *evidence of absence*.

### 3.3. H2: exploration of other playtime windows

**Figure 4.**
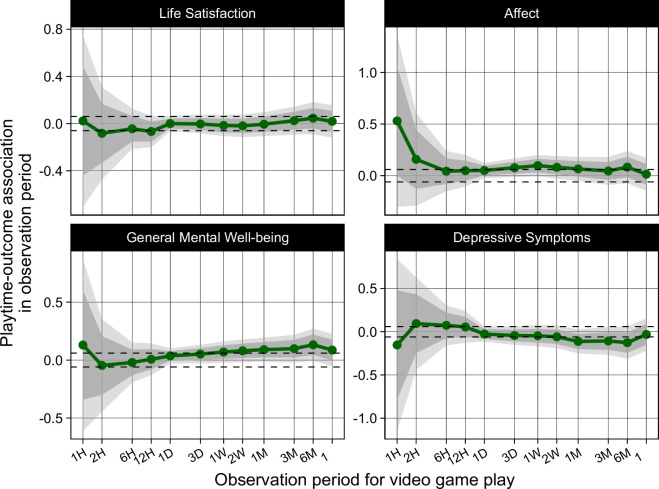
Estimates for the relationship between playtime and well-being across various timescales, shown with 90% (dark blue) and 99% (light blue) confidence intervals. Dashed lines represent the positive and negative smallest effect size of interest (SESOI) of 0.06.

Next, we conducted exploratory analyses to understand if the relationship between playtime and well-being varies across different playtime periods (figure 4). This also ensured that at least one model related playtime to well-being on a corresponding timescale (e.g. the previous two weeks for general mental well-being, and the previous one week for depressive symptomatology and and life satisfaction).

Broadly, results align with the results of H1—in all models, 99% of CIs overlapped 0, but due to low precision no estimate was fully within the equivalence bounds. We, therefore, do not find evidence for a meaningful relationship between playtime and well-being at any timescale, but cannot rule out the possibility of one existing.

Estimates are especially uncertain for observation periods of six hours or less, as only 30 participants had played Nintendo games shortly before completing the survey. However, there is a trend towards stronger relationships among more recent observation periods: based on the point estimates, playtime within the previous 1−2 h is more strongly correlated with well-being than medium- and longer-term time periods. In each case, playtime shortly before completing a survey was associated with higher affect, life satisfaction and general mental well-being; and with lower self-reported levels of depressive symptoms.

### 3.4. Exploratory analysis: moderation by life fit

Next, we conducted exploratory analyses to investigate what other factors might influence who exhibits positive or negative relations between gaming and well-being. We explored age, gender and life fit—the perceived harmful or beneficial value of gaming across various life domains outside of play. We expected that people who perceive gaming as supportive in other life domains would show positive relations between gaming and mental health, and those who perceived gaming to be harmful to other life domains would show negative ones.

To test this, we reran the models from H2, adding playtime * age, playtime * gender, and playtime * lifeFit moderation terms. We did not find evidence to support the presence of moderation; none of the moderation terms were significant (0.064 < *p* < 0.99).

**Figure 5.**
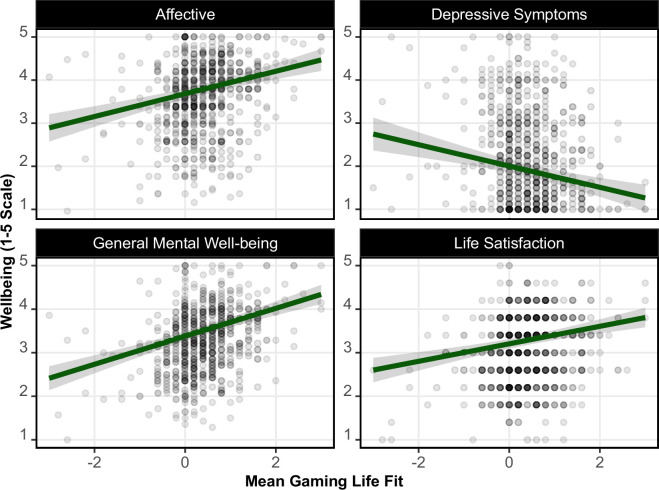
Marginal relationship between gaming life fit (perceived harmful or beneficial effects of games for oneself) and well-being.

However, we did find evidence of direct positive relationships linking life fit to well-being separate from playtime (figure 5): those who believe gaming to be beneficial to their lives tend to be also more likely to report higher levels of well-being, regardless of how much they play. Across 48 models, we observed relationships between well-being and a 1-point change in life fit ranging from 0.153 to 0.321 (median = 0.242; all *p* < 0.001). In sensitivity analyses, life fit was both a significant predictor of daily life need satisfaction (0.18; 95% CI [0.10, 0.27]) and frustration (−0.16; 95% CI [−0.27, −0.05]), and largely remained a significant predictor of well-being when adjusting for need satisfaction and frustration (median *b* = 0.111, median *p*-value = 0.014). This suggests that gaming life fit may account for well-being above and beyond someone’s level of fulfilment in life as a whole.

### 3.5. Sensitivity checks

We performed various sensitivity checks to ensure the robustness of our findings, detailed in full in the electronic supplementary material. First, we explored potential nonlinearity in the relationships between playtime and mental health by comparing a generalized additive model of well-being with and without a smooth term for playtime and comparing AIC between these. Of the 48 possible models (4 well-being variables × 12 playtime windows), just one of these (playtime in the previous one year and life satisfaction) showed a difference in AIC of more than two, indicating that nearly all relationships were adequately captured by linear terms. Next, we reran the analyses using session durations as calculated by Nintendo, as opposed to the implied duration based on the start and end timestamps; Nintendo’s durations are shorter than the implied duration in approx. 10% of sessions due to internal methodology. Data show a similar pattern: no models showed a significant relationship between playtime and well-being at our specified alpha of 0.01. Next, to address the fact that our sample was only minimally engaged with Nintendo games, we explored alternative models wherein playtime was separated into both a binary variable (1 if the player had any time logged in that period, 0 if not), and a continuous variable (how much a person played). Results were comparable; although three models indicated that among those who played in the previous 1−2 weeks, longer play is associated with higher affect and general mental well-being, the remaining 93 playtime variables were neither significant nor within both equivalence bounds. Finally, we compared models using mean scores for multi-item measures (depression and general mental well-being) to alternatives fitted using latent variable scores from a confirmatory factor analysis. Results again showed no meaningful changes; models using latent variable scores differed on average by −0.005 compared with models using mean scores.

## 4. Discussion

Although we did not intend our study to test the causal question most critical to heated debates surrounding video game engagement and global health, our study is a concrete step towards long-repeated calls for greater transparency and robustness in games research, having independently recruited a large sample of video game players (as opposed relying on recruitment through games companies themselves; e.g. Johannes *et al*. [22]), collected validated measures of well-being, joined these with digital trace data and made minimal adjustments for age, gender, employment and education. Using these methods, we did not uncover robust or consistent relationships between time spent playing and various mental well-being outcomes.

Although not conclusive, our results point toward a pattern whereby platform-wide video game play time does not predict well-being to a meaningful degree. This trend, across a wide range of outcomes, timescales of play and model specifications adds to a growing body of work that suggests that simple time spent playing games is unlikely to affect well-being for the average player. Said differently, the findings we report place the onus on those who assert that there is a meaningful relationship between playtime and well-being. It should be a priority to identify and concretely articulate which confounds might bias a true effect towards the null associations reported in this and other research using digital trace data [22,24,25,28].

To further elucidate this point, we conducted brief simulation tests to ascertain how strong such confounding might need to be (see electronic supplementary material). For example, if the true standardized effect of playtime on mental health was a moderate 0.2 s.d. value per additional hour of daily playtime, a confound C would need to be a very strong cause of both X (β=0.5) and Y (β=−0.5) to bias the true 0.2 effects to null. While we do not claim this is impossible, we do believe it unlikely. Approaching the topic along these lines—identifying confounds, testing the presence or absence of correlations for their sensitivity to potential confounds and systematically identifying factors that do (not) cause playtime and well-being—can help us achieve more systematic progress [36]. This work can be bolstered by qualitative research aimed at more fully mapping the causal system and by substantive theory development with greater specificity in the aspects of media use expected to produce effects, the hypothesized causal relationships, boundary conditions and so forth [26,37–39].

Given the growing number of studies that have failed to find meaningful relationships between playtime and well-being, the most informative such theories and models are likely to focus on the quality of play instead: the reasons why people play, the social contexts they play in, their in-game behaviour and experiences and so on. Fortunately, we have a wealth of theoretical ‘raw material’: previous research has proposed a wide variety of ways that aspects of gaming above and beyond time can affect people (see [36] for an overview of 13 such potential mechanisms). To name but a few, players may be harmed by predatory monetization or lose control over their play—phenomena more common with high playtime, but possible with lower playtime as well [40]. So too may they benefit from the development of social connections, experiences of deeper appreciation or nostalgia [41], and safe places to grapple with questions of identity [42]. We encourage the field to develop and test these theories in a systematic way, using the same rigorous methods that we have endeavoured to apply to the question of playtime and well-being.

### 4.1. Who are ‘gamers’?

The steady attrition throughout the stages of the research process from screening, to linking, to successful data retrieval highlights the challenges for participant recruitment in video game research. Despite a series of filtering steps wherein a majority of participants were filtered out due to not playing Switch games or being unwilling or unable to link data, our final sample remains only minimally engaged with Nintendo games—playing just 1.4 h per week on average. As a result, the population here is clearly different from previous studies recruiting participants with the help of game companies [22,24] or through social media forums for highly engaged players [28].

As argued above, we believe this is an important group to study in its own right, complementing the substantial research attention paid to highly engaged players (e.g. [40]). Those who rarely play video games may be particularly susceptible to their positive or negative effects on the occasions they do play. Following the Basic Needs in Games model [43] and findings that casual players typically report lower autonomy, competence and relatedness than hardcore players [44], we speculate that infrequent players may have a less accurate predictive model of how to approach and derive satisfaction from games than more seasoned players, making them more susceptible to both unexpected negative outcomes such as frustration when failing to overcome challenges or toxicity from other players, as well as positive effects such as short-term escapism, learning or social interaction. While the current study is unlikely to generalize to so-called ‘hardcore’ players who play several hours per day or more and therefore may experience more accumulative effects, our findings align with previous studies of more highly engaged players and add a new subgroup of players to the body of work showing the absence of any meaningful relationship [24,25,28].

As the field progresses, however, differences in the level of engagement pose major challenges for study sampling and generalizability. Calls for representative samples need to specify the population of reference: should this be the general population (of whom many do not play games), people who play any games at all (of whom many do not play the games for which researchers have data access), people who play the particular game or platform of interest (of whom many may be only minimally engaged) or something else entirely? In the field’s quest for more generalizable results, this will be a critical issue.

### 4.2. Timescales

While we are quick to caution that the extremely sparse data at short timescales means that we cannot draw any meaningful conclusions, our data may be able to inform future hypotheses about how quickly the effects of playtime might materialize and fade—point estimates indicated that playtime was more strongly linked with greater well-being in the 1−2 h prior to survey completion. This finding is compatible with various causal explanations: for example, players who recently played are more likely to be in a period of leisure time, which would be expected to generate more positive feelings than peers doing obligatory activities such as work. However, if researchers do expect to see positive effects of gaming, our data suggest that they may need to search for highly proximal effects directly during and following a play session (e.g. [45]).

Should future studies find effects to disapparate on similar timescales, it would go a long way towards explaining the juxtaposition of previous null findings from studies that related well-being to playtime over timescales such as 2 weeks [25], 1 month [46], 6 months [47] and 1 year [48]—alongside other studies showing small relationships over the course of a day [49,50]. For most players, it may be the case that gaming is a recovery activity that helps to manage day-to-day stresses and mood fluctuations, without necessarily having substantial long-term impacts. The majority of players have several options for activities in their environment that would have comparable effects on their well-being. Such activities are thus ‘exchangeable’, serving the same short-term goals without consequences for long-term adjustment. Studying relationships over the course of hours has to date been possible largely only in laboratory settings—rarely have researchers had access to session-level data of naturalistic behaviour that they could link to momentary well-being.

### 4.3. Life fit

This study demonstrates the potential usefulness of life fit as a theoretical construct [43]. Given the accumulating evidence that playtime and well-being are not meaningfully related at the population level alongside incontrovertible evidence that some players benefit and some are harmed [36], the task for the field can be framed as a search for the most important moderators. Life fit—a player’s self-assessment about the contribution of gaming to different aspects of their lives—stands as an effective starting point, letting researchers trust the lived experiences of players to guide them towards patterns of problematic or particularly beneficial play.

Using this measure, we found no evidence that life fit moderated the relationship between playtime and well-being, but we did find a direct correlation between the two. Notably, this relationship was an order of magnitude stronger than any estimates for playtime itself. Among several other possible explanations, this would fit a pattern of biased self-assessment: it is possible that players who are generally feeling poorly are more likely to appraise their gaming as harmful to their mental health, regardless of whether that mechanism actually takes place. This would align with some previous findings that more depressed people tend to overestimate their smartphone use due to a negative or guilt-laden appraisal process distinct from the media use itself [51].

We caution that the measure applied here has not been validated, and is better viewed as a formative indicator than as a true latent variable. More work will be needed to understand the validity of this construct.

### 4.4. Holistic digital trace data

This paper demonstrates both the value and difficulty of collecting holistic digital trace data: by capturing data across an entire platform, rather than just one game, we can potentially account for a person’s complete engagement with games without self-report biases—but only if we sample players for whom that platform constitutes the majority of their gaming. Our screening data indicate that participants play games on average across 2.8 platforms, for example playing games across Nintendo, Steam and iOS. To fully capture players’ entire gaming diets, researchers will need to either subsample participants who use only one platform or develop distinct methods of collecting digital trace data for several platforms.

In this same vein, although collaborations between academics and digital media platforms are becoming incrementally more common [24] (e.g. [52]), these remain difficult to source and stubbornly inequitably distributed across the research ecosystem. Researchers are actively exploring other ways to source digital trace data, including through scraping methods [28], APIs (e.g. https://gameplay.science), and subject data access requests/data download packages [53], but more needs to be done. Relationships between games firms and independent research teams are not scalable and the providence of data collected by scraping and related tools is difficult if not impossible to verify. Democratizing researcher access in a way that protects participants’ autonomy and right to privacy will require the enactment of multisector-spanning initiatives like the UK’s Video Game Research Framework [54] that clearly prescribe the responsibilities for those enabling, enacting and benefiting from the scientific study of video game play. The time for this is well past due.

### 4.5. Limitations

There are four limitations and constraints on the generalizability of the merit mentioned. First, we could not analyse digital trace data generated when players engaged with third-party titles (i.e. games not published by Nintendo or its closely associated companies). As a result, our findings only hold for similar Nintendo games (75% of which were rated for everyone or everyone over 10), and it is not possible to rule out the possibility that third-party games with different content or themes might show a different pattern of effects.

Second, we have access only to Nintendo Switch data, and not to other platforms or devices. While our sample is only casually engaged with Nintendo Switch games, we lack the information to know whether they are actually highly engaged players—just only on other platforms. We know that participants use on average 2.8 platforms, but we do not know how much they play on each. This is a crucial limitation to be rectified in future research that either combines digital trace data from multiple platforms or combines trace data with self-report to triangulate holistic play.

Likewise, because we collaborated with Nintendo of America, our sample consisted only of adults living in the United States, a group that we found to be largely casually involved with first-party Nintendo games. Games are both global and played by those of all ages, so it is not clear the degree to which our findings do or do not apply to younger players, those who play other games, or those who approach games from different cultural and linguistic backgrounds.

Finally, while we have longitudinal digital trace data, our self-report survey was cross-sectional, and undertaken with a view towards future more informative designs such as daily diary and experience sampling methods [49,50,55] (e.g. [56]).

## 5. Conclusion

The idea that time spent playing is the key ingredient in how games impact well-being will be with us for some time. Although our study was designed to test an association rather than a causal link, it challenges the notion that simply playing more affects well-being, for better or for worse. The correlations we observed were mostly too small to practically matter. Moreover, we show that profound confounding would be required to suppress a true causal effect to account for the null associations we report. This is improbable but not impossible, and we believe our results agree with calls for scholars and health practitioners to embrace the gradual shift towards focusing on the quality, rather than quantity of video game play as the key factor for player health—in the first instance, this might be as simple as asking players how they feel gaming fits into and differentially affects other aspects of their life such as work/school, sleep, emotion regulation, and so on. This can be done in the context of self-report questionnaires, qualitative research, diagnostic interviews, and conversations between players and loved ones. To parlay this feedback from players into more systematic theory development, we encourage researchers to familiarize themselves with the breadth of proposed mechanisms through which different patterns—rather than hours—of gaming can affect people (e.g. [36]).

By advancing generalizable accounts of how the quality of play affects well-being and simultaneously improving data quality and access, the field stands to make major strides towards our goal of not just understanding the complex relationships between video game play and well-being, but providing coherent and evidence-based solutions to improve players’ lives.

## Data Availability

This study was registered on the Open Science Framework (https://osf.io/sjqyt). Data and materials are available at OSF [57]. Data for this study were provided by Nintendo of America. Nintendo of America had no role in the design of the study, the analysis of the data, or decision to publish any results.
